# High incidence of post-operative infection after ‘sinus tarsi’ approach for treatment of intra-articular fractures of the calcaneus: a 5 year experience in an academic level one trauma center

**DOI:** 10.1186/s13037-015-0065-6

**Published:** 2015-06-02

**Authors:** Nathaniel Rawicki, Ryan Wyatt, Nicholas Kusnezov, Enes Kanlic, Amr Abdelgawad

**Affiliations:** Texas Tech Health Sciences Center, Paul L Foster School of Medicine, El Paso, TX USA

**Keywords:** Sinus tarsi, deep infection, complication, minimal invasive, intra-articular, calcaneus fracture

## Abstract

**Background:**

The optimal management of displaced intra-articular calcaneal fractures remains a topic of debate among trauma surgeons. The purpose of this study was to assess the safety of the sinus tarsi approach in regard to the incidence of deep infection and amputation following open reduction and internal fixation intra-articular calcaneal fractures.

**Methods:**

We conducted a retrospective chart review of all patients with displaced intra-articular calcaneus fractures treated with internal fixation through the sinus tarsi approach in a five year period. All surgeries were performed in a single level one trauma center by a single orthopedic trauma fellowship trained surgeon.

**Results:**

Seventeen patients with an average age of 36.6 ± 13.6 years (range 12–61 years) met the inclusion criteria. The time between injury and surgery was on average 6.1 days (range 1–22 days). Average follow up was 116 ± 78.2 days (range 3–276 days). Two patients (11.7%) had diabetes mellitus. None of the patients required amputation. Three patients (17.6%) developed deep infection and underwent subsequent formal irrigation and debridement, two of these requiring multiple repeat surgeries in addition to hardware removals. Negative pressure wound therapy and long term antibiotics via peripherally inserted central catheter (PICC) were necessary in these three patients with wound infections.

**Conclusion:**

The sinus tarsi approach for intra fixation intra-articular calcaneal fractures is safe as compared to the traditional extensile approach in regard to flap necrosis and amputation. However, the rate of deep infection was higher than previously described in the literature.

**Electronic supplementary material:**

The online version of this article (doi:10.1186/s13037-015-0065-6) contains supplementary material, which is available to authorized users.

## Introduction

Calcaneal fractures are the most common fractures of the tarsal bones with 75% being displaced and intra-articular. Intra-articular fractures of calcaneus are fractures which involve the subtalar joint [1,2]. The optimal management of displaced intraarticular calcaneal fractures has remained a controversial topic since the 19^th^ century [3]. Open reduction and internal fixation using a conventional plate via an extensile lateral, L-shaped, approach was adopted recently as the standard for treatment of these fractures [4-6]. This extensile approach provides excellent visualization and allows for direct reduction of the posterior facet fragment [7]. Though this technique has been used frequently, several studies report high postoperative wound complications using this method including wound dehiscence, flap necrosis, deep infection and even amputation of the extremity (2.0 - 19.7% ) [5,8,9]. In attempt to limit these complications, minimally invasive approaches have been proposed, including the sinus tarsi approach which allows visualization of the articular reduction while limiting soft tissue dissection [10-14].

The purpose of this study was to assess the safety and wound complications of the sinus tarsi approach in regard to the incidence of deep infection and amputation following internal fixation of displaced intraarticular calcaneal fractures.

## Methods

We conducted a retrospective chart review of all patients at a level one trauma center with displaced intraarticular calcaneal fractures treated with internal fixation through the sinus tarsi approach. All research performed with the approval of the El Paso Institutional Review Board for the Protection of Human Subjects, IRB ##00009945, and is in compliance with the Helsinki Declaration. All patients were treated by a single trauma fellowship trained surgeon. We excluded patients with extra-articular calcaneus fractures, DIACFs treated with percutaneous intervention, surgical intervention utilizing an approach other than the sinus tarsi approach (eg. extensile lateral approach), or with non-surgical intervention. Of the 33 patients treated for DIACFs during the 5 years reviewed for this study, 17 met the inclusion criteria (51.5%). The patients underwent preoperative radiographic assessment that included standard plain radiographs and CT scans as appropriate. We collected data including the gender, age, comorbidities, time from injury to surgery, total follow-up, and complications to include wound dehiscence, infection, and need for secondary surgery or amputation. A basic statistical analysis was utilized to determine mean, standard deviation, range and percentage as deemed applicable.

## Results

During 5 years study period, 17 patients between 12 and 61 years of age were surgically treated using the sinus tarsi approach. The sinus tarsi cohort included 17 patients with an average age of 36.6 ± 13.6 years (range 12–61 years). Two patients (11.7%) had diabetes mellitus. The average follow-up was 116 ± 78.2 days (range 3–276 days), with three patients having less than one month of follow-up and then were lost for follow up after that. The average follow up excluding these three patients is 141.4 days.

**Figure 1:**
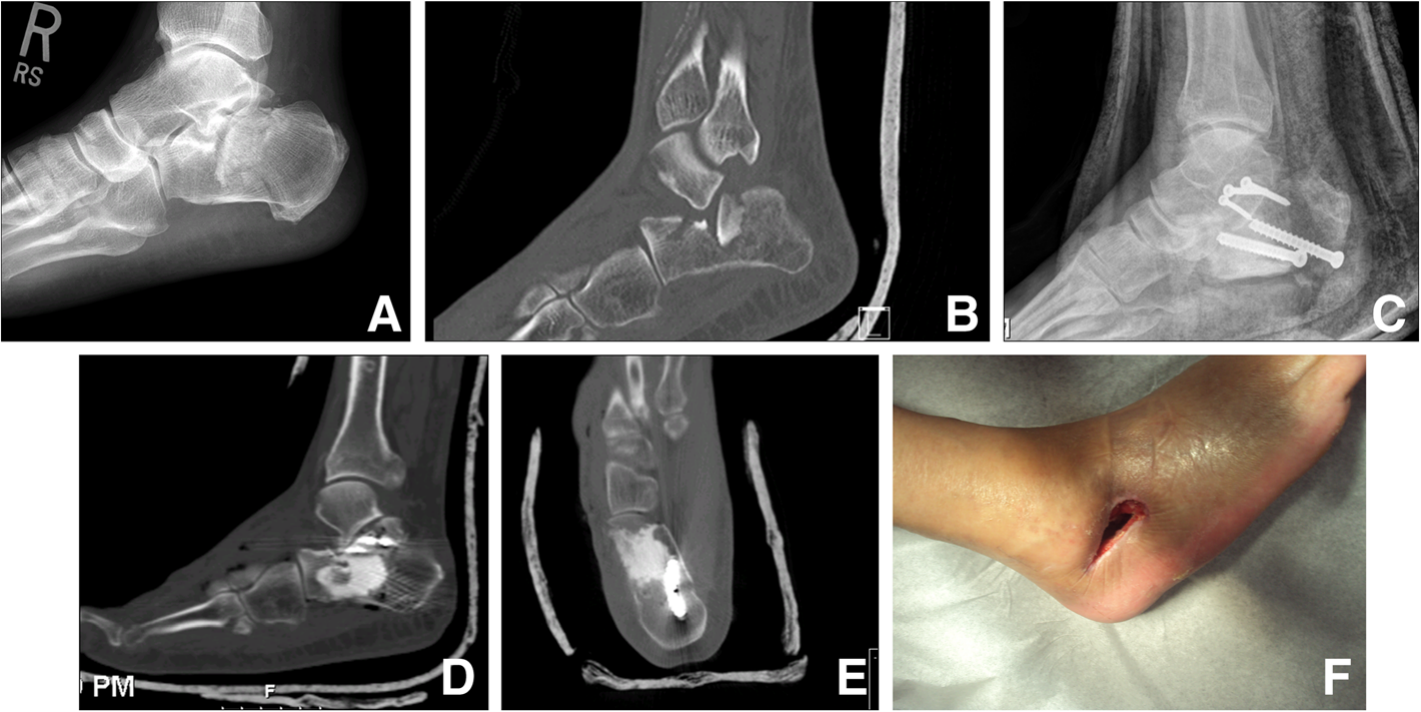
61 years old male, who had right calcaneus fracture with depression of the intra articular joint. **A, B** the injury radiograph and Sagittal CT showing the injury with severe comminution and depression. Two days after the injury, patient had open reduction using sinus tarsi approach with internal fixation using two cannulated 6.5 mm screws from the inferior aspect of the calcaneus tuberosity and three cortical screws supporting the depressed subtalar fragment. Synthetic bone grafting by Calcium Phosphate was used to fill the gap after elevating the depression. **C, D** and **E**: post operative radiograph, sagittal and coronal CT showing the reduction, fixation and grafting of the fracture. At the three weeks follow up, the patient presented with wound dehiscence and infection. Surgical debridement was performed and negative pressure wound therapy was applied until the wound healed with secondary intention to allow for regeneration of the deep defect. **F**: shows wound dehiscence under treatment by negative pressure wound therapy.

Deep wound infections occurred in three patients (17.6%), two of which were diabetic. The three patients with deep infection required subsequent irrigation and debridement in the operating room. One patient required only a single irrigation and debridement, while the other two patients required two and three repeat irrigation and debridements respectively, in addition to both requiring hardware removal. Negative pressure wound therapy was necessary to obtain wound coverage for these three patients (Figure 1) and was utilized for an average of 2.5 months.

Intra-operative deep wound cultures grew methicillin-sensitive *staphylococcus aureus* in two patients (were treated with Zosyn and Rifampin) and *Serratia marcescens* in the third (treated with Ceftriaxone).

All three patients eventually healed their infection and none of these patients went on to amputation.

## Discussion

Displaced intra-articular calcaneal fractures are relatively common high energy fractures of the lower extremity and are often managed with internal fixation through an extensile lateral incision [11,15-17]. The extensile lateral approach offers optimal exposure of fractures of the lateral wall of the calcaneus and the subtalar joint as well as direct reduction of the subtalar and calcaneocuboid articulations [3]. Despite careful surgical technique using large, well vascularized flaps, the complication rates of the extensile lateral approach remain high with the potential for deep wound infection [8,18,12]. The rate of post-operative wound infection for this approach has been cited from 2.0-19.7% [3,5,8,12,15] while the rate of general wound complications has been reported as high as 29% [3,8,12,15]. Factors contributing to the high rate of infection include concomitant medical co-morbidities, injury pattern [19], tenuous vascularity of the heel soft tissue, and the relatively large amount of soft tissue dissection involved in the extensile lateral approach [8,12].

In attempt to avoid these wound complications, several minimally invasive options have been introduced including the sinus tarsi approach [10-13,15,18]. A prospective, randomized, controlled trial by Shengli et al. comparing the radiographic outcomes of 117 displaced intra-articular calcaneal fractures, 59 of which were treated through the sinus tarsi approach and 49 via the extensile lateral approach, found no significant differences in critical radiographic measurements including change in calcaneal height, width, length, Bohler’s, and Gissane’s angles. None of the 59 patients on whom the sinus tarsi approach was used in this study developed wound complications, while 16.3% of patients who underwent open reduction internal fixation via the extensile lateral approach developed complications including dehiscence in 6 cases, wound edge necrosis in 2 cases, and secondary incision infection in 2 cases. The authors concluded that the sinus tarsi approach is safer than the extensile lateral approach, demonstrating satisfactory outcomes in the absence of a high rate of the postoperative complications [3].

Kline et al. found similar results in a retrospective review of 112 displaced intra-articular calcaneal fractures. Of the 33 which were treated through the sinus tarsi approach, 6.6% required repeat surgery for infection and 94% of patients were satisfied versus 20% and 84% respectively in the extensile lateral approach cohort [8].

Affirming the safety of the approach, in a review of 271 displaced intra-articular calcaneal fractures treated through the sinus tarsi approach, Schepers et al. found the rates of major (deep infections, osteomyelitis) and minor wound complications (wound dehiscence, superficial infection) to be 0.7% and 4.1% respectively. The authors found the functional outcomes and complication rates to be significantly better than those of the extended lateral approach, with an overall wound complication rate of 4.8% for the sinus tarsi approach and 6.6–19.7% for the extended lateral approach [10]. The low complication rate found in these studies [3,9,10] was not reproduced by our series which had a clinically higher rate of infection and unplanned surgeries (17.6%).

Kikuchi et al. reported on 22 displaced intra-articular calcaneal fractures that had open reduction and internal fixation using the sinus tarsi approach, and found a similar restoration of Böhler’s angle and calcaneal width when compared to the literature regarding the extensile lateral approach. Of the 22 fractures, 3 (13.6%) developed a superficial wound infection, and one patient required revision surgery for symptomatic hardware removal. There were no instances of deep infection or osteomyelitis. The authors concluded that the use of the sinus tarsi approach was safe and effective in the treatment of displaced intra-articular calcaneal fractures [12].

Zhang et al. in a recent article found a higher complication rate than that which had been previously described in literature. The authors compared the use of the sinus tarsi approach with another minimally invasive longitudinal approach made over the posterior part of the lateral aspect of the hindfoot along the lateral border of the Achilles tendon in 130 patients, 72 of which were fixed through the sinus tarsi approach. The sinus tarsi approach had a 12.5% rate of wound complications compared to the 2.9% incidence of the minimally invasive longitudinal approach. The wound complications included five superficial wound infection (9%) treated with dressing changes, two deep wound infection (3%) (needed surgery) and two edge necrosis (3%) (one of them needed hardware removal) [13].

Few studies have reported on the incidence of amputation secondary to infection in the setting of operatively fixed displaced intraarticular calcaneus fractures. A retrospective review of 36 patients with displaced comminuted intra articular calcaneal fractures by Siebert et al. [20] found that 5 (13.9%) necessitated amputation at an average of 44 weeks post-operatively. Conversely, Harvey and associates [18] assessed the morbidity of open reduction and internal fixation of 218 intra-articular calcaneal fractures utilizing the lateral and reported only one (0.5%) neuropathic patient who necessitated amputation. Therefore, while the incidence of amputation secondary to infection has been found to vary widely, our findings with the sinus tarsi approach are consistent with those of Harvey et al. and uphold that amputation is generally an uncommon outcome of operatively fixed displaced intra-articular calcaneal fractures.

Our results are most comparable to those of Zhang et al. [13] with even higher complication rate regarding deep wound infection. In our cohort, 3 of the 17 (17.6%) patients developed post-operative deep wound infections necessitating at least one return to the operating room per patient for formal irrigation and debridement and two hardware removals. This may be attributed to differences in patient populations. The two diabetic patients in our series developed infection. However, despite the increased rate of deep infection, none of our patients required to have an amputation for wound healing or eradication of infection.

Risk factors for infection and post-operative wound complications following operative fixation of displaced intra-articular calcaneal fractures include mainly diabetes, increased body mass index and smoking [9,21]. However, the majority of literature investigating the sinus tarsi approach does not specifically address the outcomes amongst the diabetic subset [10]. A retrospective review by Schlepers et al. [10] noted only that the one amputation was performed on a patient with peripheral vascular disease and diabetes mellitus. However the prevalence of diabetes amongst the cohort was not mentioned. Similarly, Shengli et al. [22] found no complications among 40 displaced intra-articular calcaneal fractures fixed utilizing the sinus tarsi approach, noting that two of the patients in the study had well controlled diabetes mellitus. Again, the overall prevalence of diabetes in the cohort was not specified. The high complication rate noted among diabetic patients in our study calls attention to the paucity of data on risk factors for post-operative complications and infections using the sinus tarsi approach.

The weakness of our study is mainly related to the small number of patients included and relatively short follow up period. However, this short follow up should not interfere with the rate of post operative wound infection which happens in the first few weeks after surgery.

## Conclusion

In conclusion, though the minimally invasive sinus tarsi approach did not result in flap necrosis or amputation in any of our patients, there was nevertheless a relatively high incidence of post-operative wound infection in comparison to previously reported figures. Patients should therefore be specifically counseled preoperatively of this risk. The incidence of deep infection of this approach varies significantly between different studies, suggesting that a larger patient cohort would be required to better assess the true incidence of this complication. Patients with diabetes mellitus are at higher incidence of deep infection with this approach and may be better treated with percutaneous or non-operative treatment.

## Consent

Written informed consent was obtained from the patient for the publication of this report and any accompanying images.
